# New limiting variants of the classical reiteration theorem for the *K*-interpolation method

**DOI:** 10.1186/s13660-018-1638-6

**Published:** 2018-02-20

**Authors:** Irshaad Ahmed, Maryam Ashfaq, Ghulam Murtaza

**Affiliations:** Department of Mathematics, Government College University, Faisalabad, Pakistan

**Keywords:** 46B70, 26D15, 47B38, *K*-functional, Limiting *K*-interpolation methods, Reiteration theorems, Weighted inequalities, The Fourier transform

## Abstract

We establish some reiteration theorems for limiting *K*-interpolation methods, thereby obtaining new limiting variants of the classical reiteration theorem. An application to the Fourier transform is given.

## Introduction

Let $(A_{0},A_{1})$ be a compatible couple of quasi-normed spaces, that is, we assume that both $A_{0}$ and $A_{1}$ are continuously embedded in the same Hausdorff topological vector space. Peetre’s *K*-functional $K(t,f)=K(t,f;A_{0},A_{1})$ is defined, for each $f\in A_{0}+A_{1}$ and $t>0$, by

$$K(t,f)=\inf\bigl\{ \Vert f_{0} \Vert _{A_{0}}+t \Vert f_{1} \Vert _{A_{1}}: f_{0} \in A_{0}, f_{1} \in A_{1}, f=f_{0}+f_{1}\bigr\} . $$

Let $0<\theta<1$ and $0< q\leq\infty$. The classical *K*-interpolation space $\bar{A}_{\theta,q}=(A_{0}, A_{1})_{\theta,q}$ is formed by all those $f \in A_{0}+A_{1}$ for which the quasi-norm

$$\Vert f \Vert _{\bar{A}_{\theta,q}}= \biggl( \int_{0}^{\infty}t^{-\theta q} K^{q}(t,f) \frac{dt}{t} \biggr)^{1/q} $$

is finite (here and in the sequel, the integral should be replaced by the supremum when $q=\infty$). We refer to [1–3] for the properties of the scale $\bar{A}_{\theta,q}$.

The classical reiteration theorem states that (see [4])

1.1 $$ (\bar{A}_{\theta_{0}, q_{0}}, \bar{A}_{\theta_{1}, q_{1}})_{\theta,q}= \bar {A}_{\eta,q}, $$

where $0<\theta_{0}<\theta_{1}<1$, $0<\theta<1$, $0< q_{0},q_{1},q\leq\infty$, and $\eta=(1-\theta)\theta_{0}+\theta\theta_{1}$. Moreover, for extreme cases, we have

1.2 $$ (\bar{A}_{\theta_{0}, q_{0}},{A}_{1})_{\theta,q}= \bar{A}_{\delta,q} $$

and

1.3 $$ ({A}_{0}, \bar{A}_{\theta_{1}, q_{1}})_{\theta,q}= \bar{A}_{\theta\theta_{1},q}, $$

where $\delta=(1-\theta)\theta_{0}+\theta$.

It is not hard to verify that the scale $\bar{A}_{\theta,q}$ does not make sense if $\theta=0,1$ and $q< \infty$. Let *b* be a slowly varying function (see, for instance, [5]), then the *K*-interpolation space $\bar{A}_{\theta,q;b}=(A_{0}, A_{1})_{\theta,q;b}$ consists of those $f \in A_{0}+A_{1}$ for which the quasi-norm

$$\Vert f \Vert _{\bar{A}_{\theta,q;b}}= \biggl( \int_{0}^{\infty}t^{-\theta q}b^{q}(t) K^{q}(t,f)\frac{dt}{t} \biggr)^{1/q} $$

is finite. Note that $\bar{A}_{\theta,q;b}=\bar{A}_{\theta,q}$ when $b\equiv1$. An important feature of this extended scale $\bar {A}_{\theta,q;b}$ is that it is well defined for limiting values $\theta =0,1$, under certain appropriate conditions on *b* (see [5, Proposition 2.5]). See [5–7] for different reiteration theorems for this extended scale in limiting cases. The results in these papers generalize the earlier results in [8] and [9], where the case when *b* is a broken logarithmic function was treated. In the papers [10–12], similar reiteration theorems have been derived for the more extended scale $\bar{A}_{{\theta ,b, E}}$, which is obtained by replacing the Lebesgue space $L^{q}$ ($q\geq1$) by an arbitrary rearrangement invariant normed space *E*.

Recently, Cobos et al. [13] have defined two new scales of limiting *K*-interpolation spaces $\bar{A}_{0,q;K}=(A_{0}, A_{1})_{0,q;K}$ and $\bar{A}_{0,q;K}=(A_{0}, A_{1})_{1,q;K}$, corresponding to the limiting values $\theta=0,1$, without using the extra function *b*. Namely, $\bar {A}_{0,q;K}$ and $\bar{A}_{1,q;K}$ consist of elements $f \in A_{0}+A_{1}$ with the following finite quasi-norms:

$$ \Vert f \Vert _{\bar{A}_{0,q;K}}= \biggl( \int_{0}^{1}K^{q}(t,f)\frac{dt}{t} \biggr) ^{1/q}+\sup_{t\geq1}K(t,f) $$

and

$$ \Vert f \Vert _{\bar{A}_{1,q;K}}=\sup_{0< t\leq1} \frac{K(t,f)}{t}+ \biggl( \int _{1}^{\infty} \biggl(\frac{K(t,f)}{t} \biggr)^{q}\frac{dt}{t} \biggr) ^{1/q}, $$

respectively. The main purpose in the paper [13] was to investigate the connection between these limiting *K*-interpolation methods and the interpolation over the unit square. Let us mention that these limiting *K*-interpolation methods were earlier considered in [14] and [15] in the case when the underlying couple $(A_{0},A_{1})$ is ordered, meaning that $A_{0}$ (or $A_{1}$) is continuously embedded in $A_{1}$ (or $A_{0}$). Henceforth, for the sake of simplicity, we denote $\bar{A}_{0,q:K}$ by $\bar{A}_{\{0\},q}$ and $\bar {A}_{1,q:K}$ by $\bar{A}_{\{1\},q}$.

The main goal of the present paper is to obtain limiting variants of the classical reiteration formula (1.1) by characterizing the following limiting reiteration spaces:

$$(\bar{A}_{\theta_{0}, q_{0}}, \bar{A}_{\theta_{1}, q_{1}})_{\{0\},q} $$

and

$$(\bar{A}_{\theta_{0}, q_{0}}, \bar{A}_{\theta_{1}, q_{1}})_{\{1\},q}. $$

In addition, we establish similar limiting variants of the reiteration formulae (1.2) and (1.3). In particular, Theorem 3.6 (see below) extends the assertion of [14, Theorem 4.1].

The key ingredients of our proofs will be the two-sided Hardy-type inequalities involving power-type weights. These inequalities are derived in Section 2. The main results are contained in Section 3, whereas an application to the Fourier transform is given in Section 4.

Throughout the paper, we will write $A\lesssim B$ or $B\gtrsim A$ for two non-negative quantities *A* and *B* to mean that $A\leq c B$ for some positive constant *c* which is independent of appropriate parameters involved in *A* and *B*. We put $A\approx B$ if $A \lesssim B$ and $A \gtrsim B$.

## Weighted Hardy-type inequalities

To prove our main results, we shall need suitable two-sided Hardy-type inequalities involving power-type weights. We derive them from the following general weighted Hardy-type inequalities.

### Theorem 2.1

([6], Lemma 3.2)

*Let* $0 < s < \infty$, *and assume that* *w* *and* *ϕ* *are non*-*negative functions on* $(0,\infty)$. *Put*

$$ v(t)=\bigl(w(t)\bigr)^{1-s} \biggl( \phi(t) \int_{t}^{\infty}w(u)\,du \biggr) ^{s}. $$

*Then*

2.1 $$ \int_{0}^{\infty} \biggl( \int_{0}^{t}\phi(u)h(u)\,du \biggr) ^{s}w(t)\,dt \gtrsim \int_{0}^{\infty}h^{s}(t)v(t)\,dt \quad\textit{if } 0< s< 1 $$

*and*

2.2 $$ \int_{0}^{\infty} \biggl( \int_{0}^{t}\phi(u)h(u)\,du \biggr) ^{s}w(t)\,dt\lesssim \int_{0}^{\infty}h^{s}(t)v(t)\,dt \quad\textit{if } 1 \leq s< \infty $$

*hold for all non*-*negative functions* *h* *on* $(0,\infty)$.

The next assertion deals with the general weighted Hardy-type inequality restricted to non-increasing functions.

### Theorem 2.2

([6], Lemma 3.3)

*Let* $0< s<1$ *and* $0\leq a < \infty$, *and assume that* *w* *and* *ϕ* *are non*-*negative functions on* $(0,\infty)$. *Put*

$$ v_{0}(t)=\phi(t) \biggl( \int_{0}^{t}\phi(u)\,du \biggr) ^{s-1} \int _{t}^{\infty }w(u)\,du. $$

*Then*

2.3 $$ \int_{a}^{\infty} \biggl( \int_{0}^{t}\phi(u)h(u)\,du \biggr) ^{s}w(t)\,dt\lesssim \int_{a}^{\infty}h^{s}(t)v_{0}(t)\,dt $$

*holds for all non*-*negative and non*-*increasing functions* *h* *on* $(a,\infty)$.

### Remark 2.3

The previous assertion is proved in [6] for $a=0$, but the same proof also works for all $0< a < \infty$.

Next, using the previous two theorems, we obtain the needed two-sided Hardy-type inequalities.

### Corollary 2.4

*Let* $0< s, \alpha, \beta< \infty$, *then*

$$ \int_{1}^{\infty}t^{-\alpha} \biggl( \int_{0}^{t}u^{\beta}h(u)\frac {du}{u} \biggr)^{s}\frac{dt}{t}\approx \int_{1}^{\infty}t^{\beta s-\alpha} h^{s}(t) \frac{dt}{t} $$

*holds for all non*-*negative and non*-*increasing functions* *h* *on* $(1,\infty)$.

### Proof

The estimate “≳” is a simple consequence of the fact that *h* is non-increasing. For $1\leq s <\infty$, the converse estimate “≲” follows from Theorem 2.1, applied with $w(t)=t^{-\alpha-1} \chi_{(1,\infty)}(t)$ and $\phi(t)=t^{\beta-1}$ so that $v(t)\approx t^{\beta s-\alpha-1} \chi_{(1,\infty)}(t)$. When $0< s<1$, the desired converse estimate results from Theorem 2.2, applied with $a=1$, $w(t)=t^{-\alpha-1}$, and $\phi(t)=t^{\beta-1}$, so that $v_{0}(t)\approx t^{\beta s-\alpha-1}$. The proof is complete. □

### Corollary 2.5

*Let* $0< s, \alpha, \beta< \infty$, *then*

$$ \int_{0}^{1}t^{\alpha} \biggl( \int_{t}^{\infty}u^{-\beta}g(u)\frac {du}{u} \biggr)^{s}\frac{dt}{t}\approx \int_{0}^{1}t^{\alpha-\beta s } g^{s}(t) \frac{dt}{t} $$

*holds for all non*-*negative and non*-*decreasing functions* *g* *on* $(0,1)$.

### Proof

The proof follows by applying Corollary 2.4 to the non-increasing function $h(t)=g(1/t)$ on $(1,\infty)$. □

## Limiting reiteration theorems

In this section, we establish our main results. First we need to introduce *K*-interpolation spaces of type $\mathcal{L}$ and $\mathcal {R}$. Namely, let *v* and *w* be non-negative and locally integrable functions on $(0,\infty)$, and let $0< p,q\leq\infty$. Then the *K*-interpolation spaces $\bar{A}^{\mathcal {L}}_{w,p;v,q}=(A_{0},A_{1})^{\mathcal{L}}_{w,p;v,q}$ and $\bar{A}^{\mathcal {R}}_{w,p;v,q}=(A_{0},A_{1})^{\mathcal{R}}_{w,p;v,q}$ consist of elements $f \in A_{0}+A_{1}$ with the following finite quasi-norms:

$$ \Vert f \Vert _{\bar{A}^{\mathcal{L}}_{w,p;v,q}}= \biggl( \int_{0}^{\infty }v^{q}(t) \biggl( \int_{0}^{t}w^{p}(u)K^{p}(u,f) \frac{du}{u} \biggr) ^{q/p}\frac{dt}{t} \biggr) ^{1/q} $$

and

$$ \Vert f \Vert _{\bar{A}^{\mathcal{R}}_{w,p;v,q}}= \biggl( \int_{0}^{\infty }v^{q}(t) \biggl( \int_{t}^{\infty}w^{p}(u)K^{p}(u,f) \frac{du}{u} \biggr) ^{q/p}\frac{dt}{t} \biggr) ^{1/q}, $$

respectively. For details on $\bar{A}^{\mathcal{L}}_{w,p;v,q}$ and $\bar {A}^{\mathcal{R}}_{w,p;v,q}$, the reader is referred to [6] where slightly different notations are used for these spaces. The spaces $\mathcal{L}$ and $\mathcal{R}$, with weights involving logarithmic functions or, more generally, slowly varying functions, have also appeared in [5, 8, 16]. It is also worthy of mention that the spaces $\mathcal{L}$ and $\mathcal{R}$ have been considered in [10–12] in the more general framework of rearrangement invariant spaces.

Recall that the quasi-norm on the intersection $X\cap Y$ of two quasi-normed spaces *X* and *Y* is given by

$$\Vert f \Vert _{X\cap Y} =\max\bigl( \Vert f \Vert _{X} , \Vert f \Vert _{Y} \bigr). $$

The next assertion gives a limiting variant of the reiteration formula (1.1) corresponding to the limiting value $\theta=0$.

### Theorem 3.1

*Let* $0< q_{0}, q_{1}, q < \infty$, *and* $0< \theta_{0} <\theta_{1}< 1$. *Then*

$$ (\bar{A}_{\theta_{0},q_{0}}, \bar{A}_{\theta_{1},q_{1}})_{\{0\},q}=\bar {A}_{\theta_{0},q_{0}} \cap\bar{A}^{\mathcal{L}}_{w,q_{0};v,q}, $$

*where* $w(t)=t^{-\theta_{0}}$ *and* $v(t)=\chi_{(0,1)}(t)$.

### Proof

Let $\bar{A}= (\bar{A}_{\theta_{0},q_{0}}, \bar{A}_{\theta _{1},q_{1}})_{\{0\},q} $, and take $f\in A_{0}+A_{1}$. According to [4, Theorem 2.1], we have the following formula for the classical *K*-interpolation method:

$$\begin{aligned} K\bigl(t^{\theta_{1}-\theta_{0}},f;\bar{A}_{\theta_{0},q_{0}}, \bar{A}_{\theta _{1},q_{1}}\bigr) \approx{}& \biggl( \int_{0}^{t}u^{-\theta_{0} q_{0}}K^{q_{0}}(u,f) \frac{du}{u} \biggr)^{1/q_{0}} \\ &{}+ t^{\theta_{1}-\theta_{0}} \biggl( \int_{t}^{\infty}u^{-\theta_{1} q_{1}}K^{q_{1}}(u,f) \frac{du}{u} \biggr)^{1/q_{1}}. \end{aligned}$$

Therefore, it turns out that

3.1 $$ \Vert f \Vert _{\bar{A}}\approx I_{1}+I_{2}+I_{3}+I_{4}, $$

where

$$\begin{aligned} &I_{1}= \biggl( \int_{0}^{1} \biggl( \int_{0}^{t}u^{-\theta _{0}q_{0}}K^{q_{0}}(u,f) \frac{du}{u} \biggr)^{q/{q_{0}}} \frac{dt}{t} \biggr)^{{1}/{q}}, \\ &I_{2} = \biggl( \int_{0}^{1}t^{(\theta_{1}-\theta_{0})q} \biggl( \int _{t}^{\infty}u^{-\theta_{1}q_{1}}K^{q_{1}}(u,f) \frac{du}{u} \biggr)^{{q}/{q_{1}}}\frac{dt}{t} \biggr)^{{1}/{q}}, \\ &I_{3} = \sup_{t\geq1} \biggl( \int_{0}^{t}u^{-\theta _{0}q_{0}}K^{q_{0}}(u,f) \frac{du}{u} \biggr)^{{1}/{q_{0}}} \end{aligned}$$

and

$$\begin{aligned} I_{4} = \sup_{t\geq1}t^{\theta_{1}-\theta_{0}} \biggl( \int_{t}^{\infty }u^{-\theta_{1}q_{1}}K^{q_{1}}(u,f) \frac{du}{u} \biggr)^{{1}/{q_{1}}}. \end{aligned}$$

Clearly,

3.2 $$ I_{3}= \biggl( \int_{0}^{\infty}u^{-\theta_{0}q_{0}}K^{q_{0}}(u,f) \frac{du}{u} \biggr)^{{1}/{q_{0}}}. $$

Next, let us estimate each of $I_{2}$ and $I_{4}$. We observe the simple fact that $t\mapsto K(t,f)$ is non-decreasing and apply Corollary 2.5, with $s=q/{q_{1}}$, $\alpha=\theta_{1}-\theta_{0}$, $\beta=\theta _{1}q_{1}$, and $g(t)=K^{q_{1}}(t,f)$, to get

3.3 $$ I_{2} \approx \biggl( \int_{0}^{1}t^{-\theta_{0}q}K^{q}(t,f) \frac {dt}{t} \biggr)^{{1}/{q}}. $$

Moreover, noting the fact that $t\mapsto t^{-1}K(t,f)$ is non-increasing, we have

$$\begin{aligned} I_{1} &\geq \biggl( \int_{0}^{1}t^{-q}K^{q}(t,f) \biggl( \int _{0}^{t}u^{(1-\theta_{0})q_{0}} \frac{du}{u} \biggr)^{q/{q_{0}}} \frac {dt}{t} \biggr)^{{1}/{q}} \\ &\approx \biggl( \int_{0}^{1}t^{-\theta_{0}q}K^{q}(t,f) \frac{dt}{t} \biggr)^{{1}/{q}} \end{aligned}$$

whence, in view of (3.3), we get $I_{1} \gtrsim I_{2}$. Therefore,

3.4 $$ I_{1}+I_{2} \approx I_{1}. $$

Now we estimate $I_{4}$. Observe that

$$\begin{aligned} I_{4} &\leq \sup_{t\geq1}t^{\theta_{1}-\theta_{0}} \biggl( \int _{t}^{\infty}u^{(\theta_{0}-\theta_{1})q_{1}}\frac{du}{u} \biggr)^{{1}/{q_{1}}}\cdot\sup_{t\geq1}t^{-\theta_{0}}K(t,f) \\ &\approx \sup_{t\geq1}t^{-\theta_{0}}K(t,f). \end{aligned}$$

In addition, we have

$$\biggl( \int_{t}^{\infty}u^{-\theta_{0}q_{0}}K^{q_{0}}(u,f) \frac {du}{u} \biggr)^{{1}/{q_{0}}} \gtrsim t^{-\theta_{0}}K(t,f),\quad t>0, $$

as $t \mapsto K(t,f)$ is non-decreasing. Combining the previous two estimates, we arrive at

$$I_{4}\lesssim \biggl( \int_{1}^{\infty}u^{-\theta_{0}q_{0}}K^{q_{0}}(u,f) \frac{du}{u} \biggr)^{{1}/{q_{0}}}. $$

This, along with (3.2), leads us to

3.5 $$ I_{3}+I_{4} \approx \biggl( \int_{0}^{\infty}u^{-\theta _{0}q_{0}}K^{q_{0}}(u,f) \frac{du}{u} \biggr)^{{1}/{q_{0}}}. $$

Now inserting estimates (3.4) and (3.5) in (3.1) yields

$$\begin{aligned} \Vert f \Vert _{\bar{A}} &\approx \biggl( \int_{0}^{1} \biggl( \int_{0}^{t}u^{-\theta_{0}q_{0}}K^{q_{0}}(u,f) \frac{du}{u} \biggr)^{q/{q_{0}}} \frac{dt}{t} \biggr)^{{1}/{q}}+ \biggl( \int _{0}^{\infty}t^{-\theta_{0}q_{0}}K^{q_{0}}(t,f) \frac{dt}{t} \biggr)^{{1}/{q_{0}}} \\ &= \Vert f \Vert _{\bar{A}^{\mathcal{L}}_{w,q_{0};v,q}}+ \Vert f \Vert _{\bar{A}_{\theta_{0},q_{0}}}, \end{aligned}$$

which finishes the proof. □

Next we establish a limiting version of the reiteration formula (1.1) corresponding to the limiting value $\theta=1$.

### Theorem 3.2

*Let* $0< q_{0}, q_{1}, q < \infty$, *and* $0< \theta_{0} <\theta_{1}< 1$. *Then*

$$ (\bar{A}_{\theta_{0},q_{0}}, \bar{A}_{\theta_{1},q_{1}})_{\{1\},q}=\bar {A}_{\theta_{1},q_{1}} \cap\bar{A}^{\mathcal{R}}_{w,q_{1};v,q}, $$

*where* $w(t)=t^{-\theta_{1}}$ *and* $v(t)=\chi_{(1,\infty)}(t)$.

### Proof

In view of the following elementary identity:

3.6 $$ K(t,f;A_{0},A_{1})=tK\bigl(t^{-1},f;A_{1},A_{0} \bigr),\quad t>0, $$

the symmetry property $(A_{0},A_{1})_{\{0\},q}=(A_{1},A_{0})_{\{1\},q}$ holds. Together with the well-known symmetry property for the scale $\bar {A}_{\theta,q}$, this gives

$$(\bar{A}_{\theta_{0},q_{0}}, \bar{A}_{\theta_{1},q})_{\{1\},q}= \bigl((A_{1}, A_{0})_{1-\theta_{1},q}, (A_{1},A_{0})_{1-\theta_{0},q_{0}} \bigr)_{\{0\},q}. $$

Now applying Theorem 3.1 yields

$$\begin{aligned} &\Vert f \Vert _{(\bar{A}_{\theta_{0},q_{0}}, \bar{A}_{\theta_{1},q_{1}})_{\{1\} ,q}}\\ &\quad\approx \biggl( \int_{0}^{1} \biggl( \int_{0}^{t}u^{(\theta _{1}-1)q_{1}}K^{q_{0}}(u,f;A_{1},A_{0}) \frac{du}{u} \biggr)^{q/{q_{1}}} \frac {dt}{t} \biggr)^{{1}/{q}} \\ &\qquad{}+ \biggl( \int_{0}^{\infty}t^{(\theta _{1}-1)q_{1}}K^{q_{1}}(t,f;A_{1},A_{0}) \frac{dt}{t} \biggr)^{{1}/{q_{1}}} \\ &\quad= \biggl( \int_{0}^{1} \biggl( \int_{0}^{t}u^{\theta _{1}q_{1}}K^{q_{1}} \bigl(u^{-1},f\bigr) \frac{du}{u} \biggr)^{q/{q_{1}}} \frac {dt}{t} \biggr)^{{1}/{q}} \\ &\qquad{}+ \biggl( \int_{0}^{\infty}t^{\theta_{1}q_{1}}K^{q_{0}} \bigl(t^{-1},f\bigr)\frac {dt}{t} \biggr)^{{1}/{q_{1}}} \\ &\quad= \biggl( \int_{1}^{\infty} \biggl( \int_{t}^{\infty}u^{-\theta _{1}q_{1}}K^{q_{1}}(u,f) \frac{du}{u} \biggr)^{q/{q_{1}}} \frac{dt}{t} \biggr)^{{1}/{q}} \\ &\qquad{}+ \biggl( \int_{0}^{\infty}t^{-\theta_{1}q_{1}}K^{q_{1}}(t,f) \frac {dt}{t} \biggr)^{{1}/{q_{1}}} \\ &\quad = \Vert f \Vert _{\bar{A}^{\mathcal{R}}_{w,q_{1};v,q}}+ \Vert f \Vert _{\bar{A}_{\theta_{1},q_{1}}}, \end{aligned}$$

which completes the proof. □

The next two results provide limiting variants of the reiteration formula (1.2) corresponding to the limiting values $\theta=0,1$.

### Theorem 3.3

*Let* $0< q_{0}, q < \infty$, *and* $0< \theta_{0} <1$. *Then*

$$ (\bar{A}_{\theta_{0},q_{0}}, A_{1})_{\{0\},q}= \bar{A}_{\theta_{0},q_{0}} \cap\bar{A}^{\mathcal{L}}_{w,q_{0};v,q}, $$

*where* $w(t)=t^{-\theta_{0}}$ *and* $v(t)=\chi_{(0,1)}(t)$.

### Proof

Let $\bar{A}=(\bar{A}_{\theta_{0},q_{0}}, A_{1})_{\{0\},q}$, and take $f\in A_{0}+A_{1}$. Applying the following formula (see [4, Remark 2.1]):

3.7 $$ K\bigl(t^{1-\theta_{0}},f;\bar{A}_{\theta_{0},q_{0}}, A_{1} \bigr) \approx \biggl( \int _{0}^{t}u^{-\theta_{0} q_{0}}K^{q_{0}}(u,f) \frac{du}{u} \biggr)^{1/q_{0}}, $$

we immediately have

$$\Vert f \Vert _{\bar{A}}\approx \Vert f \Vert _{\bar{A}^{\mathcal{L}}_{w,q_{0};v,q}}+ \Vert f \Vert _{\bar{A}_{\theta_{0},q_{0}}}, $$

which completes the proof. □

### Theorem 3.4

*Let* $0< q_{0}, q < \infty$, *and* $0< \theta_{0} < 1$. *Then*

$$(\bar{A}_{\theta_{0},q_{0}}, A_{1})_{\{1\},q}= \bar{A}_{\{1\},q}. $$

### Proof

Let $\bar{A}=(\bar{A}_{\theta_{0},q_{0}}, A_{1})_{\{1\},q}$, and take $f\in A_{0}+A_{1}$. Then, making use of formula (3.7), we have

3.8 $$ \Vert f \Vert _{\bar{A}}=I_{1}+I_{2}, $$

where

$$I_{1}=\sup_{0< t \leq1} t^{\theta_{0}-1} \biggl( \int_{0}^{t}u^{-\theta _{0}q_{0}}K^{q_{0}}(s,f) \frac{du}{u} \biggr)^{{1}/{q_{0}}} $$

and

$$I_{2}= \biggl( \int_{1}^{\infty}t^{(\theta_{0}-1)q} \biggl( \int _{0}^{t}u^{-\theta_{0}q_{0}}K^{q_{0}}(u,f) \frac{du}{u} \biggr)^{{q}/{q_{0}}} \frac{dt}{t} \biggr)^{{1}/{q}}. $$

Applying Corollary 2.4, with $s={q}/{q_{0}}$, $\beta=(1-\theta _{0})q_{0}$, $\alpha=(1-\theta_{0})q$, and $h(t)=t^{-q_{0}} K^{q_{0}}(t,f)$, gives

3.9 $$ I_{2}\approx \biggl( \int_{1}^{\infty} \biggl(\frac{K(t,f)}{t} \biggr)^{q} \frac {dt}{t} \biggr)^{{1}/{q}}. $$

Next we estimate $I_{1}$. We have

$$\begin{aligned} I_{1} &\leq \sup_{0< t\leq1}t^{\theta_{0}-1} \biggl( \int_{0}^{t}u^{(1-\theta _{0})q_{0}}\frac{du}{u} \biggr)^{{1}/{q_{0}}}\cdot\sup_{0< t\leq1 } \frac{K(t,f)}{t} \\ &\approx \sup_{0< t\leq1 } \frac{K(t,f)}{t}. \end{aligned}$$

Also, since $t \mapsto t^{-1}K(t,f)$ is non-increasing, we have

$$\begin{aligned} I_{1} \geq \sup_{0< t\leq1}t^{\theta_{0}-2}K(t,f) \biggl( \int _{0}^{t}u^{(1-\theta_{0})q_{0}}\frac{du}{u} \biggr)^{{1}/{q_{0}}} \approx \sup_{0< t\leq1 } \frac{K(t,f)}{t}. \end{aligned}$$

Thus,

3.10 $$ I_{1}\approx\sup_{0< t \leq1} \frac{K(t,f)}{t}. $$

Inserting estimates (3.9) and (3.10) in (3.8) completes the proof. □

Using the same symmetry argument as in the proof of Theorem 3.2, we can derive the following limiting variants of the reiteration formula (1.3) from the previous two theorems.

### Theorem 3.5

*Let* $0< q_{1}, q < \infty$, *and* $0< \theta_{1}< 1$. *Then*

$$ ({A}_{0}, \bar{A}_{\theta_{1}, q_{1}})_{\{1\},q}= \bar{A}_{\theta _{1},q_{1}} \cap\bar{A}^{\mathcal{R}}_{w,q_{1};v,q}, $$

*where* $w(t)=t^{-\theta_{1}}$ *and* $v(t)=\chi_{(1,\infty)}(t)$.

### Theorem 3.6

*Let* $0< q_{1}, q < \infty$, *and* $0 <\theta_{1}< 1$. *Then*

$$({A}_{0}, \bar{A}_{\theta_{1},q_{1}})_{\{0\},q}= \bar{A}_{\{0\},q}. $$

### Remark 3.7

If $A_{1}$ is continuously embedded in $A_{0}$, then Theorem 3.6 gives back [14, Theorem 4.1].

## An application

The Fourier transform $\mathcal{F}$ of a function $f \in L^{1}(\mathbb {R}^{n})$ is defined as

$$\mathcal{F}[f](\xi)= \int_{\mathbb{R}^{n}}f(x)e^{-i\xi\cdot x}\,dx,\quad \xi\in\mathbb{R}^{n}. $$

The next result provides an application of Theorem 3.4 to the mapping properties of the Fourier transform. For related results, the reader is referred to the papers [17, 18], and [5] and a recent PhD dissertation [19]. As usual, let $f^{\ast}$ denote the non-increasing rearrangement (see, for instance, [3]) of *f*. Put $f^{\ast\ast}(t)=\int_{0}^{t}f^{\ast}(u)\,du, t>0$.

### Theorem 4.1

*Let* $0< q<\infty$, *and set*

$$E= \biggl\{ f\in L^{1}\bigl(\mathbb{R}^{n} \bigr)+L^{\infty}\bigl(\mathbb{R}^{n}\bigr): \Vert f \Vert _{E}= \Vert f \Vert _{L^{1}(\mathbb{R}^{n})}+ \biggl( \int_{0}^{1}t^{q} f^{\ast\ast}(t)^{q} \frac {dt}{t} \biggr)^{1/q}< \infty \biggr\} $$

*and*

$$F= \biggl\{ f\in L^{1}\bigl(\mathbb{R}^{n} \bigr)+L^{\infty}\bigl(\mathbb{R}^{n}\bigr): \Vert f \Vert _{F}= \Vert f \Vert _{L^{\infty}(\mathbb{R}^{n})}+ \biggl( \int_{1}^{\infty} f^{\ast}(t)^{q} \frac {dt}{t} \biggr)^{1/q}< \infty \biggr\} . $$

*Then* $\mathcal{F}$ *is bounded as a map from* *E* *to* *F*.

### Proof

We simply put $L^{1}=L^{1}(\mathbb{R}^{n})$ and $L^{\infty}=L^{\infty }(\mathbb{R}^{n})$. It is well known that $\mathcal{F}$ is bounded as a map from $L^{1}$ to $L^{\infty}$ and also as a map from $L^{2}$ to $L^{2}$. Therefore, by the interpolation property of the *K*-interpolation method $\bar{A}_{\{1\},q}$ (see [13, Proposition 3.2]), $\mathcal{F}$ is bounded as a map from $( L^{2}, L^{1})_{\{1\},q}$ to $( L^{2}, L^{\infty} )_{\{1\},q}$. Thus, the proof is complete if we show that $( L^{2}, L^{1})_{\{1\},q}=E$ and $( L^{2}, L^{\infty} )_{\{1\},q}=F$. To this end, we observe that $(L^{1},L^{\infty})_{1/2,2}=L^{2}$ and $(L^{\infty},L^{1})_{1/2,2}=L^{2}$, and apply Theorem 3.4 to obtain that

$$\begin{aligned} \bigl( L^{2}, L^{1} \bigr)_{\{1\},q}&= \bigl( \bigl(L^{\infty},L^{1}\bigr)_{1/2,2}, L^{1} \bigr)_{\{1\},q} \\ &=\bigl( L^{\infty}, L^{1}\bigr)_{\{1\},q} \\ &=\bigl(L^{1}, L^{\infty}\bigr)_{\{0\},q} \end{aligned}$$

and

$$\begin{aligned} \bigl( L^{2}, L^{\infty} \bigr)_{\{1\},q}&= \bigl( \bigl(L^{1},L^{\infty}\bigr)_{1/2,2}, L^{1} \bigr)_{\{1\},q} \\ &=\bigl( L^{1}, L^{\infty}\bigr)_{\{1\},q}. \end{aligned}$$

It remains to show that $(L^{1}, L^{\infty})_{\{0\},q}=E$ and $( L^{1}, L^{\infty})_{\{1\},q}= F$. Let $f\in L^{1}+L^{\infty}$, then (see [1, Theorem $5.2.1$])

$$K\bigl(t,f;L^{1}, L^{\infty}\bigr)= \int_{0}^{t}f^{\ast}(u)\,du,\quad t>0. $$

Thus,

$$\begin{aligned} \Vert f \Vert _{(L^{1}, L^{\infty})_{\{0\},q}}&= \biggl( \int_{0}^{1} \biggl( \int _{0}^{t}f^{\ast}(u)\,du \biggr)^{q} \frac{dt}{t} \biggr)^{1/q}+ \sup _{t\geq 1} \int_{0}^{t}f^{\ast}(u)\,du \\ &= \biggl( \int_{0}^{1}t^{q} f^{\ast\ast}(t)^{q} \frac{dt}{t} \biggr)^{1/q} + \int_{0}^{\infty}f^{\ast}(u)\,du \\ &= \Vert f \Vert _{E} \end{aligned}$$

and

$$\begin{aligned} \Vert f \Vert _{(L^{1}, L^{\infty})_{\{1\},q}}&= \sup_{0< t\leq1} \frac{1}{t} \int _{0}^{t}f^{\ast}(u)\,du+ \biggl( \int_{1}^{\infty}t^{-q} \biggl( \int _{0}^{t}f^{\ast}(u)\,du \biggr)^{q} \frac{dt}{t} \biggr)^{1/q} \\ &= f^{\ast}(0) + \biggl( \int_{1}^{\infty}t^{-q} \biggl( \int_{0}^{t}f^{\ast}(u)\,du \biggr)^{q} \frac{dt}{t} \biggr)^{1/q} \\ &\approx f^{\ast}(0)+ \biggl( \int_{1}^{\infty} f^{\ast}(t)^{q} \frac {dt}{t} \biggr)^{1/q}, \end{aligned}$$

where the last equivalence follows from Corollary 2.4, applied with $s=q$, $\beta=1$, $\alpha=q$, and $h=f^{\ast}$. Hence, $\Vert f \Vert _{(L^{1}, L^{\infty})_{\{1\},q}}\approx \Vert f \Vert _{F}$. The proof is complete. □
